# Addressing the Antimicrobial Resistance of Ruminant Mycoplasmas Using a Clinical Surveillance Network

**DOI:** 10.3389/fvets.2021.667175

**Published:** 2021-06-14

**Authors:** Maryne Jaÿ, François Poumarat, Adélie Colin, Agnès Tricot, Florence Tardy

**Affiliations:** ^1^UMR Mycoplasmoses animales, Anses, Université de Lyon, Lyon, France; ^2^UMR Mycoplasmoses animales, VetAgro Sup, Université de Lyon, Marcy-l'Étoile, France

**Keywords:** antimicrobial resistance, surveillance, mycoplasma, ruminant, method standardization

## Abstract

Antimicrobial resistance (AMR) surveillance of mycoplasmas of veterinary importance has been held back for years due to lack of harmonized methods for antimicrobial susceptibility testing (AST) and interpretative criteria, resulting in a crucial shortage of data. To address AMR in ruminant mycoplasmas, we mobilized a long-established clinical surveillance network called “Vigimyc.” Here we describe our surveillance strategy and detail the results obtained during a 2-year monitoring period. We also assess how far our system complies with current guidelines on AMR surveillance and how it could serve to build epidemiological cut-off values (ECOFFs), as a first attainable criterion to help harmonize monitoring efforts and move forward to clinical breakpoints. Clinical surveillance through Vigimyc enables continuous collection, identification and preservation of *Mycoplasma* spp. isolates along with metadata. The most frequent pathogens, i.e., *M. bovis* and species belonging to *M. mycoides* group, show stable clinicoepidemiological trends and were included for annual AST. In the absence of interpretative criteria for ruminant mycoplasmas, we compared yearly minimum inhibitory concentration (MIC) results against reference datasets. We also ran a SWOT (Strengths, Weaknesses, Opportunities, Threats) analysis on the overall service provided by our AMR surveillance strategy. Results of the 2018–2019 surveillance campaign were consistent with the reference datasets, with *M. bovis* isolates showing high MIC values for all antimicrobial classes except fluoroquinolones, and species of the *Mycoides* group showing predominantly low MIC values. A few new AMR patterns were detected, such as *M. bovis* with lower spectinomycin MICs. Our reference dataset partially complied with European Committee on Antimicrobial Susceptibility Testing (EUCAST) requirements, and we were able to propose tentative epidemiological cut-off values (TECOFFs) for *M. bovis* with tilmicosin and spectinomycin and for *M. mycoides* group with tilmicosin and lincomycin. These TECOFFs were consistent with other published data and the clinical breakpoints of *Pasteurellaceae*, which are often used as surrogates for mycoplasmas. SWOT analysis highlighted the benefit of pairing clinical and antimicrobial resistance surveillance despite the AST method-related gaps that remain. The international community should now direct efforts toward AST method harmonization and clinical interpretation.

## Introduction

Despite being mostly non-transmissible to humans, mycoplasmas, i.e., bacteria belonging to the *Mycoplasma* genus, encompass animal pathogens of major importance, responsible for huge economic losses in food-producing animals worldwide (1). *Mycoplasma* diseases are mostly controlled using antimicrobials (2). The ruminants sector is underserved with effective vaccines (3, 4), which makes it doubly essential to preserve antimicrobial efficacy. Antimicrobial resistance (AMR) surveillance programs are key to the process, as they provide both early warning about potential emergence and a starting basis for adapting treatments in response to changes in resistance patterns (5, 6). Surveillance relies on robust, standardized and internationally harmonized antimicrobial susceptibility testing (AST). However, the complex *in vitro* growth requirements of mycoplasmas mean that AST cannot be performed using the most common routinizable method of disk diffusion. AMR data for mycoplasmas consequently lagged behind other bacteria for years, particularly in veterinary species, until a first robust AST methodology based on minimal inhibitory concentration (MIC) determination was proposed in 2000 (7). However, 20 years later, there is still a need to better standardize veterinary mycoplasma AST by developing harmonized methodologies and interpretative criteria, i.e., clinical breakpoints (CBP) set by standardization institutes like CLSI (Clinical & Laboratory Standards Institute) or EUCAST (European Committee on Antimicrobial Susceptibility Testing) (2). CBP are hard to establish in veterinary medicine as they need to be species-specific, substance-specific, and disease-specific (8). Nonetheless, CBP are essential to promoting prudent and appropriate antimicrobial use as part of a wider “antimicrobial stewardship” concept factoring in AMR trends (9). CBP are missing for many combinations of animal/bacteria species and many clinical conditions. Epidemiological cut-off values (ECOFFs), i.e., the highest MIC that defines the upper end of the wild-type (WT) MIC distribution, could be a first step toward CBP determination. ECOFFs provide thresholds for early warning of acquired phenotypical resistance, allowing the distinction between WT and non-WT isolates and, hence, supporting surveillance programs (10). However, ECOFFs are less straightforward than CBP for guiding therapy, as non-WT isolates are not always clinically resistant. Furthermore, the process of setting ECOFFs is also a challenge, as according to EUCAST standard operating procedures, it requires aggregation of MIC data obtained in different laboratories using standardized AST methods (11). Consequently, at a level below ECOFFs, tentative ECOFFs (TECOFFs) defined without meeting with all EUCAST requirements, could valuably inform the surveillance of mycoplasmas AMR (11).

AMR surveillance programs have various objectives, such as (i) to describe AMR trends as “a rational basis for establishing empirical therapy, and for evaluating and comparing strategies to counteract the development of resistance,” (ii) to serve as “an inspiration for standardization and harmonization of antimicrobial susceptibility testing among laboratories” and (iii) to detect new antimicrobial resistance patterns and mechanisms in order to help interpret AST (6).

AMR surveillance programs are increasingly being developed in the animal sector, but with a clear priority on zoonotic pathogens and indicator commensals (9) anchored in “One Health” concept. Animal-only pathogens like mycoplasmas have remained under-investigated for years, especially at international level (5, 12, 13), despite accounting for a large share of antimicrobial use in livestock. In France, plans established by the French Ministry of Agriculture and Food (EcoAntibio Plan I and II) to counter AMR managed to reduce antibiotic use by 37% between 2012 and 2016^1^ but not to extend AMR surveillance to bacteria that have so far been neglected. The well-established French AMR surveillance system “Resapath” that collates AST data from collaborating veterinary laboratories generates AMR surveillance data on both zoonotic and non-zoonotic pathogenic bacteria of animal origin but still does not include bacteria like mycoplasmas that escape AST using the standard disk diffusion method (12, 14).

Several studies have contributed to a first mapping of ruminant mycoplasma AMR in different parts of the world [see (2) for review]. However, most of the data have been generated using different methodologies, and only a few studies give an overview of AMR evolution over time in a defined geographic area (15–19). The challenge in developing AMR surveillance in veterinary mycoplasmas is therefore to define a framework allowing continuous systematic collection and analysis of AMR data, and ultimately elements to help interpret these data in order to guide the choice of antimicrobials for clinical therapy (8).

Vigimyc is a passive clinical surveillance network focused on ruminant mycoplasmoses in France. It has been operating since 2003 with different objectives as detailed previously (20). Vigimyc surveillance is grounded in the continuous identification of clinical isolates sourced from culture-based diagnoses performed in local laboratories on the initiative of veterinarians or livestock breeders. We posit that the Vigimyc network could fill in the gap in mycoplasma AMR monitoring in ruminants, as it provides a continuous collection of well-documented clinical isolates.

This paper progresses in three steps. First we describe the surveillance strategy and how it was built around routine Vigimyc operations. Second, we present and critically analyze the results of continuous monitoring. Third, we assess whether our clinical-based monitoring system can contribute to the overall improvement of AMR surveillance in ruminant mycoplasmas and the development of AST interpretative criteria.

## Materials and Methods

### Clinical Mycoplasmosis Surveillance Through Vigimyc

The Vigimyc surveillance system mobilizes a network of 35 first-line diagnosis laboratories, on average, that routinely isolate *Mycoplasma* spp.-like colonies from clinical specimens, in partnership with our laboratory that identifies *Mycoplasma* species in the pre-cultures (both broth and agar) forwarded to us by these laboratories. As a central coordinator laboratory we regularly issue and revise operating guidelines on best practice for isolating mycoplasmas from clinical specimens. We also organize wet-lab training sessions open for technicians from partner laboratories. Guidelines and training session ensure that procedures remain homogeneous throughout the network. Identification of isolates up to species or subspecies level is done mostly using dot-immunobinding on membrane filtration (MF-dot) using anti-sera prepared against representative strains of each species (21). If identification fails, then species-specific PCR or universal PCR targeting Mollicutes 16S rRNA followed by sequence analysis is used (20). The protocol detects both pathogenic and commensal/opportunistic, cultivable (sub)species.

Metadata on the clinical specimens (using a shared terms' list for host animal species, geographic origin, nature of the specimen, clinical signs, age of sampled animals and sampling date, etc.) is sent by partner laboratories and recorded in an in-house database (developed by JL Vinard using a MySQL database management system and a Windev Computer Aided Software Engineering from PCSoft to provide a user-friendly operator interface). This database allows easy detection of “duplicates,” i.e., cultures sourced from the same village or the same animal with a common sampling date.

The identification rate is the proportion of pre-cultures received that results in a positive identification of *Mycoplasma* spp. Non-identification can come from absence of mycoplasma, overgrowth of contaminants, or viability loss. To maximize our chances of identifying species mixes or slow-growing species, the MF-dot analysis is run on both the original liquid culture from the partner laboratory and a re-culture of several colonies selected from their agar plates.

A first-line preservation protocol is systematically performed by snap-freezing (at −80°C) a contaminant-free aliquot of *Mycoplasma* spp.-positive re-cultures. The preservation rate is the proportion of cultures preserved at first line. When necessary, a long-term glycerol-supplemented set of isolates is preserved at −80°C after a series of quality controls (viability, cloning for species mixes, absence of contamination, additional identification, etc.).

### Antimicrobial Susceptibility Testing

In the absence of a reference method recognized by standard-setting organizations (EUCAST and CLSI), AST was performed by determining the MICs of different antimicrobials using the agar dilution method according to guidelines for veterinary mycoplasmas (7) and as previously described (17). For each MIC assay, identical batches of PPLO agar medium (Indicia Production, Saint-Genis-L'Argentière, France) were used and a minimum of two control plates without antimicrobial were included to control for mycoplasma loads that are expected to be between 30–300 CFU per 1 μL spot to be readable. A reference strain with a known MIC profile was also added twice on each plate for quality control, as recommended (7). If the mycoplasma load on control plates or MIC of the reference strains was not as expected, the MIC series was not validated and was re-run. All antimicrobials tested were purchased from Sigma-Aldrich (Saint-Quentin-Fallavier, France).

The most frequently-diagnosed pathogenic *Mycoplasma* species evidenced through clinical surveillance were submitted to AST, i.e., *M. bovis* in cattle and species belonging to or related to the *M. mycoides* group in goats: *M. mycoides* subsp. *capri* (*Mmc*), *M. capricolum* subsp. *capricolum* (*Mcc*), and *M. putrefaciens* (*Mp*). The AMR surveillance strategy was designed to assess the putative annual evolution of the MIC distributions of current strains in comparison to a “reference” population, as a baseline for surveillance. All isolates from the reference population or included in the annual surveillance program were selected among Vigimyc isolates using the following criteria: one isolate only per sampling date and per village, distributed as widely as possible within “départements,” and with diverse associated clinical signs whenever possible. To save time in the cloning steps, cultures containing more than one *Mycoplasma* species were not retained as a first choice.

For the reference data set, MIC distribution was determined by testing the whole range of 2-fold dilutions of antimicrobials, from 0.0625 to 128 μg/mL, according to EUCAST SOP 10.1 (11) on both recent vs. older isolates. The rationale for including older isolates was to increase the possibility to detect WT isolates, i.e., isolates with no acquired resistance. One drug per class of antimicrobial was selected: tylosin or tilmicosin (macrolides), lincomycin (lincosamides), enrofloxacin (fluoroquinolones), oxytetracycline (tetracyclines), spectinomycin (aminosides), florfenicol (phenicols), according to market authorizations and therapeutic indications in ruminant hosts. The reference distributions for *M. bovis* MICs were retrieved from two previous studies conducted in our laboratory in which AST was performed on Vigimyc isolates using the same methodology (15, 17). It included between 73 and 170 MIC values, depending on the antimicrobial, that were obtained on both “recent” (between 2000 and 2014) and “older” (after 1978 and before 2000) isolates. Contrary to the situation for *M. bovis*, no internal reference dataset was available for the *M. mycoides*-group (sub)species. The reference distribution was established here by testing 58 *Mmc*, 60 *Mcc*, and 51 *Mp* isolates collected between 1977 and 2016, that is 144 “recent” (2011–2016) and 25 “older” (1977–1999) isolates. To generate this dataset, we determined the MICs of the most commonly-used antimicrobials in small ruminants (Supplementary Figure 1).

For routine annual surveillance initiated in 2018, MICs were estimated by testing only two to five concentrations of one antimicrobial per class, chosen according to MIC reference distributions and defined to capture the evolution compared to the reference population (see section Results and Discussion). This strategy aimed at sparing testing-time in order to make yearly surveillance compatible with routine practice and available resources while enabling to quicken early comparison with the reference dataset. A minimum of 10 (and up to 59) isolates per year and per species were chosen from the Vigimyc collection according to their isolation frequency, geographical diversity, clinical history and purity in initial culture (not being part of a subculture containing a mix of *Mycoplasma* species). Two in-house control strains were systematically included in the annual surveillance protocol in order to ensure inter-assay reproducibility.

### Determination of Epidemiological Cut-Offs

MIC distributions obtained with the reference datasets were tentatively used to determine ECOFFs. However, as these MICs did not fulfill all EUCAST SOP 10.1 requirements (11), we used the term “tentative ECOFF” or “TECOFF” instead of ECOFF. Whenever possible, we estimated both visual and numerical TECOFFs, using the ECOFFinder program (ECOFFinder 2.1).

## Results and Discussion

### Clinicoepidemiological Trends From the Vigimyc Network Over the 2014–2019 Period

Between 2014 and 2019, 2,982 cultures were collected through Vigimyc (Table 1). They originated from a steady yearly number of 53–60 collecting “départements.” These “départements” encompass all the main livestock areas in France, including 16 and 15 that featuring in the top 20 in terms of head of cattle and head of goats, respectively^2^. The number of cultures collected yearly over the 2014–2019 period, was on average 24% higher than during the 2008–2013 period. The identification rate was also higher in 2014–2019 (94%) vs. 2008–2013 (87%). This illustrates an increase in the capacity of partner laboratories to grow and detect mycoplasmas in culture, a know-how ensured through regular technical and theoretical training, shared guidelines, and quality control of media batches. These figures also reflect the all-round soundness of clinical monitoring with a large number of collected cultures, continued commitment of laboratories, and sustained expertise on mycoplasma diagnosis. The resulting identified isolates were mostly from cattle (annual mean *n* = 226), followed by goats (annual mean *n* = 167) and sheep (annual mean *n* = 78).

**Table 1:** Vigimyc surveillance results over the 2014–2019 period in cattle, sheep and goats, including dominant clinical signs, *Mycoplasma* species identified, and number of isolates preserved in collection.

	**2014**	**2015**	**2016**	**2017**	**2018**	**2019**	**Total 2014–2019**	**Mean 2014–2019**	**Mean 2008–2013**
**Panel A: cultures received for identification per year**									
Number of “départements” of origin	53	53	64	57	60	53	340	57	54
Number of cultures	494	534	506	434	485	529	2,982	497	380
Total identified (rate %)	479 (97%)	508 (95%)	471 (93%)	406 (93%)	466 (96%)	494 (93%)	2,824 (95%)	465 (94%)	333 (87%)
Total preserved^*^ (rate %)	430 (90%)	448 (88%)	393 (83%)	336 (90%)	425 (91%)	457 (92%)	2,489 (88%)	414 (89%)	Not known
**Panel B: clinical signs and** ***Mycoplasma*** **species in cattle**									
No. of cultures with *Mycoplasma* spp. identification	269	260	246	201	170	207	1,353	226	169
**Clinical signs**									
Respiratory	236	224	233	179	153	183	1,208	201	144
Mastitis	7	5	0	1	1	1	15	3	4
Other	8	12	4	15	14	22	75	13	10
Not known	18	19	9	6	2	1	55	9	9
***Mycoplasma*** **species**									
*M. bovis*	180	151	152	121	108	128	840	140	96
*M. bovirhinis*	107	95	83	85	62	75	507	85	67
*M. arginini*	62	64	74	34	38	45	317	53	25
*M. alkalescens*	6	14	10	10	11	13	64	11	5
Others^a^	6	16	8	3	8	13	54	9	7
**Panel C: clinical signs and** ***Mycoplasma*** **species in goats**									
No. of cultures with *Mycoplasma* spp. identification	146	187	143	124	203	198	1001	167	135
**Clinical signs**									
Respiratory	47	41	43	37	57	73	298	50	28
Mastitis	42	74	46	39	72	28	301	50	46
Arthritis	13	23	15	13	20	17	101	17	14
Association of ≥ 2 of these signs	5	13	12	9	18	15	72	12	6
Other	5	8	4	10	15	22	64	11	11
Not known	34	28	23	16	21	43	165	28	24
***Mycoplasma*** **species**									
*M. mycoides* subsp. *capri*	42	73	65	53	94	62	389	65	52
*M. capricolum* subsp. *capricolum*	41	51	53	40	44	44	273	46	33
*M. putrefaciens*	20	26	19	7	28	31	131	22	19
*M. agalactiae*	6	1	4	2	12	7	32	5	5
*M. ovipneumoniae*	15	17	7	11	16	37	103	17	4
*M. arginini*	29	20	17	29	39	61	195	33	15
Others^b^	1	3	4	5	2	3	18	3	2
**Panel D: clinical signs and** ***Mycoplasma*** **species in sheep**									
No. of cultures with *Mycoplasma* spp. identification	64	61	82	81	93	89	470	78	47
**Clinical signs**									
Respiratory	44	48	75	63	86	71	387	65	29
Other	5	6	5	7	4	14	41	7	6
Not known	15	7	2	11	3	4	42	7	10
***Mycoplasma*** **species**									
*M. ovipneumoniae*	28	21	24	43	49	36	201	34	14
*M. arginini*	46	48	71	60	76	74	375	63	33
Others^c^	4	0	3	2	5	11	25	4	5

* *Cultures with at least one species successfully preserved (viability); “Others” include:*

a *M. canadense, M. bovigenitalium, M. mycoides subsp. capri, M. ovipneumoniae, A. laidlawii, M. maculosum, M. canis, M. bovoculi;*

b *A. laidlawii, M. conjunctivae, M. bovis, M. auris, M. yeattsii, M. cottewi;*

c *M. agalactiae, M. mycoides subsp. capri, M. conjunctivae, M. bovis*.

Clinical signs recorded in the sampled animals over 2014–2019 were comparable to 2008–2013 (20). There was a dominance of respiratory signs in cattle (201 samples on average, i.e., 89%) and sheep (65 samples on average, i.e., 81%). In goats, respiratory and mammary signs were the most frequent (both averaging 50 samples, i.e., 30% each), in line with contagious agalactia (CA) syndrome (4). However, clinical metadata were missing for around 30 goat specimens each year, and so the clinical picture was not fully complete.

A vast majority of the cultures received were identified straight away using MF-dot (21), and only 2%, between 2017 and 2019, had to be submitted to other identification methods. The *Mycoplasma* species identified were mostly similar in 2008–2013 compared to 2014–2019. In cattle, *M. bovis* remained dominant (annual mean value of 140 isolates, i.e., 62% of cultures with a *Mycoplasma* spp. identified) with respiratory tropism, whereas *M. alkalescens* was rarely isolated (annual mean value of 10 isolates, 5%) like other rarely-detected species, such as *M. canadense, M. bovigenitalium, M. bovoculi, M. canis*, with a maximum of 6 isolates (4%) per year. In goats, among the four species involved in CA syndrome, *Mmc* and *Mcc* were most frequently isolated [65 (39%) and 46 (28%) samples per year on average, respectively], largely ahead of *Mp* (annual mean of 22 isolates, 13%) and *M. agalactiae* (annual mean of 5 isolates, 3%). The increase in numbers of *M. ovipneumoniae* isolates in both sheep and goats over 2008–2013 continued in the 2014–2019 period and was the only evolving trend for ruminant pathogenic mycoplasmas (21).

Beyond these pathogenic species, the frequency of opportunistic/commensal species remained stable over 2014–2019. In cattle, *M. bovirhinis* was the most frequent (annual mean of 85 isolates, 37%). *M. arginini* was also frequently isolated with 53 (23%), 63 (79%) and 33 (19%) isolates per year on average in cattle, sheep and goats, respectively. *M. bovirhinis* was associated with *M. bovis* in 19% of cases on average over 2014–2019 while *M. arginini* was associated with *M. bovis* in 66% of cases. *M. arginini* was isolated with CA-causing agents in 19% of cases in goats, and with *M. ovipneumoniae* in 10 and 31% of cases in goats and sheep, respectively (data not shown).

Besides clinical surveillance, the network also builds up a collection of clinical isolates over time, and with some even pre-dating official creation of the network, as some of the oldest isolates dated back to the late 1970s. Between 2014 and 2019, the preservation rate allowed a first-line conservation of 414 cultures out of 465 identified per year on average (Table 1). After several quality controls, isolates derived from these cultures have regularly been included in various studies on epidemiology [see for example (23)], method validation (24) or mycoplasma biology (25). In the last decade, this collection has also been a valuable source of material for studying the AMR of French isolates over time (15–17, 22). The resulting susceptibility patterns per species were very varied, with recent French *M. bovis* isolates (collected after year 2010) predominantly resistant to most antimicrobials except fluoroquinolones, while small-ruminant species such as *M. agalactiae* and *M. ovipneumoniae* have—with a few exceptions—remained far more susceptible. This underscores the need for long-term AMR monitoring programs. An initiative took shape in 2018 to start building a continuous AMR surveillance system based on Vigimyc isolates.

### AMR Surveillance Data Generated From Vigimyc Isolates

The 2018–2019 AMR surveillance was based on comparing MICs of annual isolates to that of a reference population.

#### *M. bovis*: Reference Population and Surveillance Results

Aggregated MICs distributions used as reference for the *M. bovis* population in France (15, 17) are presented in Table 2. For tilmicosin and spectinomycin, visual inspection of MICs distributions gave a clear-cut bimodal distribution, with no overlap, suggesting two different populations. The population with low MIC values (upper limit of 4 μg/mL for tilmicosin and 8 μg/mL for spectinomycin) contained exclusively older isolates (*n* = 31 and *n* = 27, respectively) and was considered as wild-type (WT). In contrast, for oxytetracycline, florfenicol and enrofloxacin, the MICs were continuously distributed, thus ruling out visual determination of upper WT limit. For enrofloxacin, Gautier-Bouchardon et al. (15) demonstrated that there were effectively two peaks, distant by a 2-fold dilution only, evidencing two overlapping populations, one composed of older isolates (*n* = 27) and centered on MIC = 0.25 μg/mL and the other composed of more recent isolates centered on MIC = 0.5 μg/mL. This distribution points to a WT vs. a non-WT population, but it was not associated with point mutations linked to resistance (17). Nonetheless, based on these distributions, we were able to choose antimicrobial concentrations to be tested for annual surveillance in order to assess any shifts in MICs compared to the recent reference isolates, i.e., 16 and 512 μg/mL for tilmicosin, 4 and 64 μg/mL for oxytetracycline, 4 and 16 μg/mL for florfenicol, 64 and 256 μg/mL for spectinomycin, and 0.25 and 1 μg/mL for enrofloxacin.

**Table 2:**
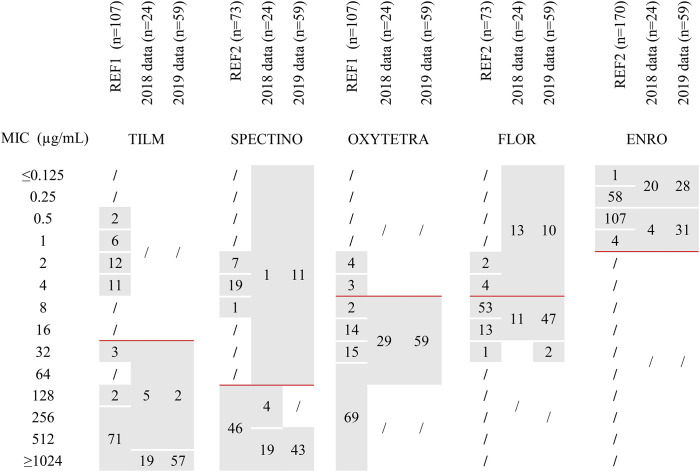
MICs distributions of 5 antimicrobials for *M. bovis* isolates either from the reference populations or from 2018–2019 annual monitoring.

*Number of isolates in each MIC category are shown. “/” means no isolate in the corresponding category. Pasteurellaceae clinical intermediate breakpoints for cattle (CLSI 2015) are represented by a horizontal red separator line. REF1 and REF2 correspond to reference datasets of isolates collected between 1978–2014 (15, 17) and 1978–2012 (15), respectively. ENRO, enrofloxacin; FLOR, florfenicol; OXYTETRA, oxytetracycline; SPECTINO, spectinomycin; TILM, tilmicosin*.

In 2018 and 2019, 24 and 59 isolates of *M. bovis*, respectively, were included in the annual AMR monitoring program (Table 2). The MICs distribution of tilmicosin tightly overlapped with the non-WT reference population, and MICs for oxytetracycline, florfenicol and enrofloxacin were distributed in a pattern consistent with the reference populations. One noticeable difference was observed for spectinomycin, where a population with MICs below 64 μg/mL was detected in 2018 (*n* = 1) and 2019 (*n* = 11) that was absent from the “recent” strains (2010–2012) of the reference dataset (15). Furthermore, between 2018 and 2019, there was an increase in isolates with very high MIC values (≥1,024 μg/mL) for tilmicosin (from 79 to 97%) and a potential shift toward higher MICs for florfenicol (from 46 to 83% of MIC ≥ 8 μg/mL) and enrofloxacin (from 17 to 52% of MIC values equal to 0.5 or 1 μg/mL). These data need to be consolidated by further similar observations over the coming years.

#### *M. mycoides* Group: Reference Population and Surveillance Results

MIC distributions of 169 isolates of the *Mycoides* group (sub)species, taken as a reference dataset, are represented in Supplementary Figure 1 and summarized in Table 3. For tylosin, we found a dominant population with low MIC values centered on 0.125 μg/mL (≤0.0625–0.25) that was putatively considered as WT, whereas 14 recent *Mcc* and 3 recent *Mmc* isolates showed increased widely-distributed MICs ranging between 1 and 128 μg/mL. The important spread of MICs suggests different, possibly cumulative mechanisms of AMR. This is completely different from *M. bovis*, for which the shift between WT and non-WT was strictly correlated with homogeneous mutations in the ribosomal target (17). For lincomycin, two slightly overlapping populations were evidenced, one dominant and centered on 2 μg/mL, and the other with MICs above 8 μg/mL observed for *Mcc* (recent and older) and *Mmc* (recent). This fairly continuous distribution ruled out an unequivocal definition of the WT upper limit (putatively between 4 and 8 μg/mL). For enrofloxacin, oxytetracycline and spectinomycin, whatever the species, the MIC distributions were monomodal, centered on 0.125, 0.5, and 64 μg/mL, respectively.

**Table 3:**
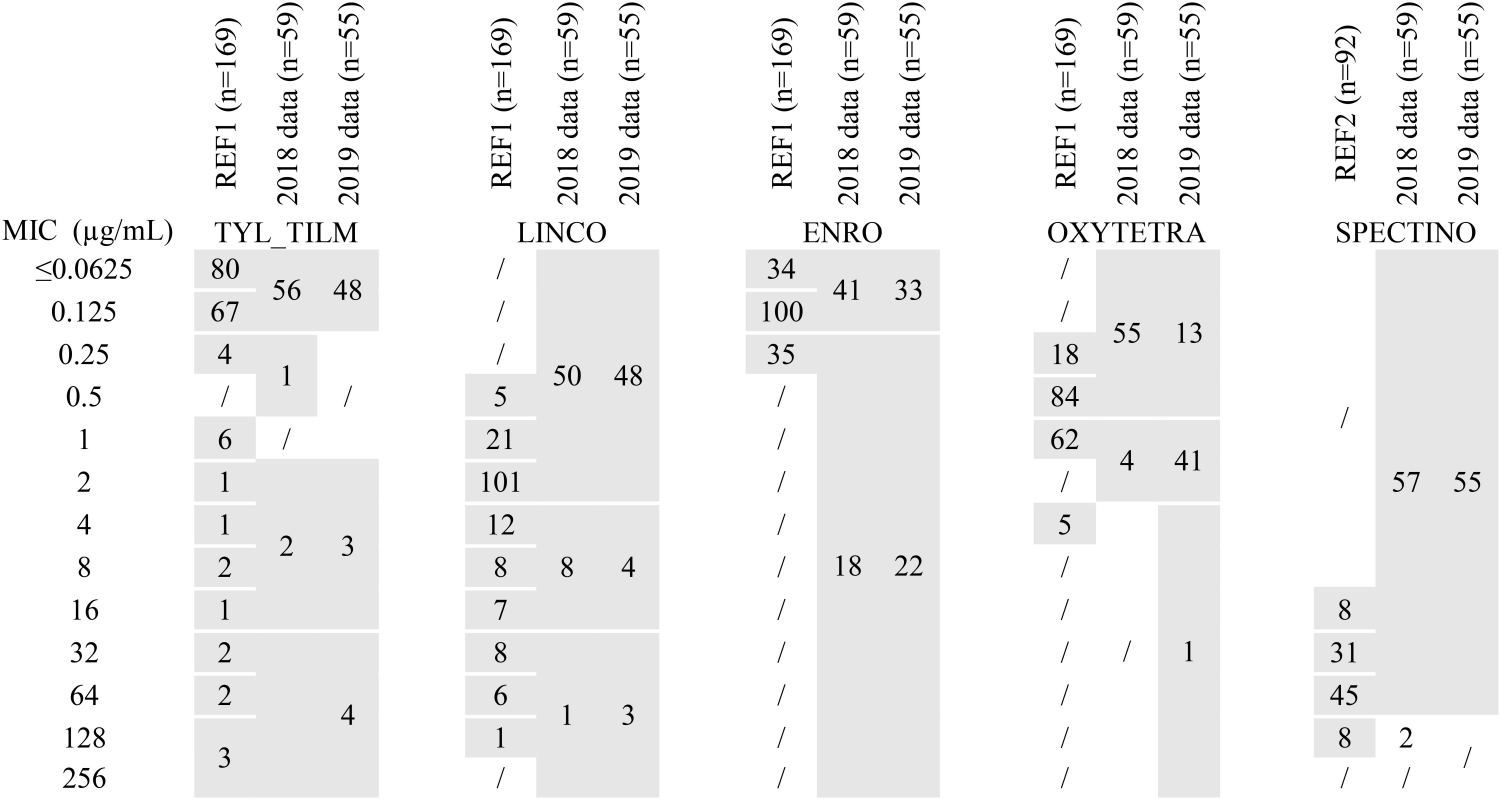
MICs distributions of 5 antimicrobials for isolates of the *M. mycoides* cluster either from the reference population or from 2018-2019 annual monitoring.

*Reference data results from aggregation of the data presented in Supplementary Figure 1, i.e., MIC distributions of “recent” and “older” population belonging to the three species M. mycoides subsp. capri, M. capricolum subsp. capricolum and M. putrefaciens. Number of isolates in each MIC category are shown. “/” means no isolate in the corresponding category. Note that for macrolides, tilmicosin was tested for annual surveillance and tylosin for the reference dataset. ENRO, enrofloxacin; LINCO, lincomycin; OXYTETRA, oxytetracycline; SPECTINO, spectinomycin; TILM, tilmicosin; TYL, tylosin*.

The three (sub)species harbor a similar distribution whatever the antimicrobial considered, except that no *Mp* isolate with increased tylosin or lincomycin MICs was observed in the reference set of strains. Furthermore, these three (sub)species are phylogenetically and genomically closely related (26, 27). Consequently, and despite not being recommended by EUCAST, we chose to aggregate MICs within the *M. mycoides* cluster as a single reference population for annual surveillance (Table 3). We consequently chose the following concentrations of antimicrobials to be tested to detect shifts from the reference population: 0.125, 1 and 16 μg/mL for tilmicosin, 0.5 and 2 μg/mL for oxytetracycline, 2 and 16 μg/mL for lincomycin, 64 and 128 μg/mL for spectinomycin, and 0.125 and 0.25 μg/mL for enrofloxacin.

In 2018 and 2019, 38 and 28 isolates of *Mmc*, 11 and 17 isolates of *Mcc* and 10 and 10 isolates of *Mp*, respectively, were included in the annual AMR monitoring program. This distribution paralleled the frequency of detection of each sub(species) within Vigimyc, although the MICs were aggregated for comparison to the reference dataset (Table 3). MICs remained unchanged in 2018 and 2019 compared to the reference population, with (i) for tylosin and lincomycin, a dominant population below 0.125 and 2 μg/mL and a few strains with MICs exceeding 1 and 16 μg/mL, respectively, (ii) for oxytetracycline and enrofloxacin, monomodal populations centered around 0.125 and 0.5 μg/mL, respectively, and (iii) for spectinomycin, a dominant population harboring MIC ≤ 64 μg/mL. In 2019, we introduced some supplementary concentrations to check the upper and lower limits. The results confirmed that higher lincomycin MICs (*n* = 3) do not exceed 128 μg/mL, that most of enrofloxacin MICs above 0.125 μg/mL were equal to 0.25 μg/mL (*n* = 22), and that spectinomycin MICs were mostly between 16 and 64 μg/mL (*n* = 53). Note, however, that two isolates from 2019 harbored spectinomycin MIC ≤ 8 μg/mL, which was not observed in the reference dataset. Unlike in the reference dataset, there were also increased tilmicosin or lincomycin MICs for Mp (one strain with a tilmicosin MIC between 2 and 16 μg/mL and one with lincomycin MIC >16 μg/mL; data not shown).

Our approach enabled to evidence putative multiresistant isolates harboring high MICs for at least 3 antimicrobials. For instance, in 2019, 4 isolates of *Mcc* had MICs ≥ 2 μg/mL for tilmicosin, ≥4 μg/mL for lincomycin and ≥32 for spectinomycin (data not shown). These multiresistant patterns were also detected in our reference dataset, and thus require particular vigilance as they could result in emergence and clonal expansion, as shown for *M. bovis* (28).

### Larger-Scale Contribution and Improvement of the Surveillance Framework

To broaden the perspectives of our surveillance framework, we assessed whether our data could contribute to the determination of ECOFFs that could usefully distinguish WT and non-WT populations or serve as a first step toward CBPs. According to EUCAST SOP, the process of setting ECOFFs requires aggregation of at least 5 datasets of different origins, each one containing (i) exact MIC values and (ii) at least 15 isolates belonging to the WT population, that needs to have a normal 3–5 dilutions-wide distribution. The size of the WT population in this aggregated set should be increased to >100 isolates for ECOFF determination using statistical approaches (EUCAST SOP). Our reference dataset was partly compliant with these criteria. We were thus able to determine some tentative ECOFFs (TECOFFs) for certain mycoplasmal species and antimicrobials. These TECOFFs were compared to those derived from other published data.

For *M. bovis*, the continuous MICs distribution for florfenicol and oxytetracycline, the truncated enrofloxacin distribution at the lower end (≤0.125 μg/mL), and the low number of WT isolates (<100) for antimicrobials with a bimodal distribution (tilmicosin, spectinomycin) ruled out any reliable statistical calculation of TECOFF. However, for tilmicosin and spectinomycin the bimodal distribution and the size and quality of the presumptive WT population (31 and 27 isolates and 4 and 3 dilutions wide, respectively) enabled us to propose visual TECOFFs of 8 μg/mL for tilmicosin and 16 μg/mL for spectinomycin. These results are consistent with Hata et al. (29) whom MIC distributions using the agar dilution method allowed us to visually set TECOFFs at 8 μg/mL for both tilmicosin and spectinomycin (*n* = 203 isolates including 26 WT and 159 WT, respectively). For spectinomycin, a statistical TECOFF calculation using ECOFFFinder was also possible due to the large number of WT isolates included in their study, thus giving us a consistent 8 μg/mL value. Another study based on the broth-dilution method consistently end up in our hands in a visual TECOFF of 16 μg/mL for spectinomycin (30), which is interesting as it highlights a limited impact of the chosen methodology. For oxytetracycline, florfenicol and enrofloxacin, the monomodal distribution evidenced on our dataset was also found in other studies (18, 19, 30–33), which rules out a TECOFF.

For the *Mycoides* group (Table 3), the situation is more favorable to both visual and statistical TECOFF determination, with lower MICs overall for most antimicrobials and hence a potential WT population. The only exception was spectinomycin which had a unique population with high MICs (>8 μg/mL), in line with other studies (MIC ≥ 8 μg/mL) (34–37) and supporting the hypothesis of an intrinsic resistance within the *M. mycoides* cluster (38, 39). The MICs distribution for tylosin showed a large WT population of 151 isolates with an upper limit of 0.25 μg/mL, but the truncated lower MIC limit (<0.0625 μg/mL) did not allow calculation of a statistical TECOFF. Other studies reported either a bimodal distribution (37, 40) or a monomodal distribution (36) with a slightly lower upper limit of the WT populations at between 0.12 μg/mL (40) and 0.2 μg/mL (36, 37). For enrofloxacin, the unique population we observed had an upper limit at 0.5 μg/mL, consistent with results from other studies, i.e., a unique population with a maximum MIC of 0.4 μg/mL (34–37, 40), but the truncated lower end again rules out a statistical TECOFF. For lincomycin, the TECOFF visually derived from our data was putatively between 4 and 8 μg/mL and was definitively set at 4 μg/mL using ECOFFinder with a 99.9% endpoint. This result slightly exceeds those obtained by Tatay et al. (37) for *Mcc*, with lincomycin MICs showing a bimodal distribution with a WT upper limit at 1.6 μg/mL. Visual and statistical TECOFFs for oxytetracycline were estimated as 2 μg/mL on our results, which is similarly slightly higher than the visual TECOFF derived from other studies (≤1μg/mL) (40, 41).

In conclusion, we were able to use our data to propose several TECOFFs (Tables 2, 3), most of which were consistent or only one to two dilutions different to other datasets, even if these were obtained using other AST methodology.

Note that our proposed TECOFFs for *M. bovis* (8 μg/mL for tilmicosin and 16 μg/mL for spectinomycin) were consistent with *Pasteurellaceae* CBP that would classify any strain with an MIC ≤8μg/mL for macrolides and ≤ 32 μg/mL for aminoglycosides as “susceptible.” *Pasteurellaceae* CBP are often used as a surrogate to interpret *M. bovis* MIC by exploiting the shared tissue tropism of the bacteria. As expected, the two proposed TECOFFs are slightly below or equal to CBPs (10). The clinical interpretation of *M. bovis* AST results, based on *Pasteurellaceae* CBPs, confirmed the exclusively resistant populations from 2018–2019 for all antimicrobials except enrofloxacin, spectinomycin and florfenicol. Furthermore, most of the isolates harbored multiresistant patterns.

A similar approach using other bacterial CBPs cannot be implemented for *M. mycoides*-group species due to the broad diversity of associated clinical signs. However, based on our MIC distributions, we can hypothesize that antimicrobial efficiency is likely to be preserved *in vivo* for enrofloxacin, tilmicosin, lincomycin, oxytetracycline except for a few cases with the 3 later antimicrobials. Besides, resistance to spectinomycin should be further explored.

### Assessment of the Service Provided by Vigimyc for AMR Surveillance

We assessed the contribution of Vigimyc to AMR surveillance according to several guidelines (5, 6, 9, 11, 42, 43), with two major focuses: one on isolates (number, qualification and relevance) and the other on methods (AST method and harmonization, choice of antimicrobial, and data collection, analysis and reporting, interpretative criteria, sustainability of the monitoring framework). This analysis was conducted using a SWOT matrix (Table 4). Strengths (S in Table 4) and weaknesses (W in Table 4) are typically considered as internal factors inherently under control of a system, but here, as we built the AMR surveillance program on a pre-existing epidemiological surveillance system, they needed to be re-analyzed. In contrast, opportunities (O in Table 4) and Threats (T, in Table 4) are external factors that need to be examined to define future perspectives for AMR surveillance. The objective of this initial assessment, after only 2 years of operation, was to identify potential improvements and promote similar initiatives from other countries that would in turn contribute to a better AMR surveillance in mycoplasmas worldwide.

**Table 4:** SWOT analysis of AMR surveillance performed through Vigimyc over the 2018-2019 period.

**Strengths**	**Weaknesses**
S1 Pre-existing frame for routine clinical surveillance = easy access to species identification and specimen metadata. S2 Sufficient number of clinical isolates per year for the most frequent pathogenic species (> 20) + possibility for multiannual data aggregation for infrequent species. S3 Geographic and clinical representativeness of enrolled isolates + steadiness of clinical epidemiological trends = continuity of AMR monitoring. S4 Easy possibility to rule out any duplicate isolates. S5 Harmonized and robust method for isolation of clinical isolates shared within the network. S6 Application of available recommended AST methods. S7 AST centralized in one laboratory = no methodological variability. S8 Common annual reporting for both clinical and AMR surveillance.	W1 For multifactorial diseases, no joint AST of different bacterial pathogens contained within the same clinical specimen. W2 Several methodological choices are done to spare time and make surveillance routine-compatible. W3 AST data for infrequent species are of limited interpretative interest if analyzed annually. W4 No interpretative criteria = limited feedback to veterinarians. W5 Limited contribution to setting ECOFFs when no obvious WT population is evidenced or because of an incomplete or truncated range of MIC tested.
**Opportunities**	**Threats**
O1 Potential expansion to other *Mycoplasma* species O2 Optimal framework to observe correlations between clinical and resistance trends. O3 Potential switch to a multicentre surveillance system with development of AST methods more adapted to routine work. O4 AMR data (reference dataset) could be helpful for setting TECOFFs and for clinical interpretation	T1 AST performed in a central laboratory and not by a consortium of laboratories = risk of a poor long-term sustainability. T2 Lack of harmonization around AST methods worldwide. T3 Switch to a molecular diagnosis of mycoplasmosis = > no more strains collected for AMR monitoring.

*Strengths [S], Weaknesses [W], Opportunities [O], and Threats [T] are numbered for reference in the main text*.

#### Isolates: Qualification, Number, and Relevance

The isolates enlisted for AST all originated from the Vigimyc surveillance and were therefore clinically relevant and identified to the species level as required by EUCAST SOP [S1, S3]. Metadata collected within the Vigimyc framework—i.e., animal, location, species, age, clinical signs, and specimen—also met the recommendations of Cornaglia et al. (6) transposed to animals [S1]. In the absence of guidelines concerning adequate quantitative sampling for surveillance, the number of tested isolates per (sub)species of interest was proportional to their relative clinical prevalence [S2]. Thus, in 2018–2019, AST was performed on 10 to 59 isolates for each target species (out of a total of between 28 and 128 isolates identified through surveillance). For the less-frequent species, two types of aggregation were possible, either multiannual or by grouping species regarded as genetically and/or phenotypically similar with a comparable risk for AMR emergence, as suggested by Cornaglia et al. (6). This sampling, although non-random, enabled the detection of new patterns, such as very recent *M. bovis* isolates being more susceptible to spectinomycin than the reference population. Looking ahead to the next few years, AMR surveillance could easily be enlarged to other collected pathogen species, *M. ovipneumoniae* (on an annual basis) and *M. agalactiae* (on a multiannual basis), as sound AST methods and reference data are available (16, 22) [O1]. AST could also be further extended to commensal/opportunist mycoplasmas that could make useful sentinel species if horizontal gene transfer is suspected.

Concerning isolates sampling, no explicit recommendation on representativeness is yet available. In our monitoring system, clinical isolates submitted to AST benefit from the broad geographical coverage, coherent with breeding areas, provided by Vigimyc and are further selected to maximize their geographical origin [S3]. For instance, in 2019, *M. bovis* and *M. mycoides* cluster isolates originated from 21 and 22 “départements,” respectively. Stable epidemiological trends mean that a similar sampling scheme can be considered for the coming years. Note that the choice of a national coverage precludes regional comparisons due to of a low number of isolates in each area.

The use of clinically-relevant isolates is often recommended (5, 6) and is ensured by the Vigimyc framework as it deals only with diseased animals [S3]. However, because some clinical signs, age classes or production types are dominant in Vigimyc-sampled animals (Table 1), they are also dominant in the AST panel. We remain alert with respect to a potential bias and for *M. bovis* we have already shown the absence of influence of (i) tissue tropism (17), or (ii) sampling time post antimicrobial therapy (23) on AMR.

The clinical surveillance data made it easy to exclude duplicate samples from the 2018–2019 panel [S4], as recommended (6, 11, 43). Furthermore, isolates originating from the same village but with different sampling dates, which are not true duplicates, were considered as second choice for AST, as they could be repeated samples in the event of persistent mycoplasmosis.

Overall, coupling clinical and AMR surveillance enables early investigation of the involvement of AMR in epidemiological trends and shifts, as already performed in dedicated studies (22, 28) [O2]. Looking at the broader perspective, despite mycoplasmas being frequently involved in multifactorial diseases, it is a pity that we are currently unable to link our AMR data with that of other bacterial pathogens collected on the same clinical specimens in order to adopt a global clinical AMR approach, as started in specific studies (23) [W1].

#### Methods: MIC Determination, Data Reporting, AST Interpretation

Isolation was performed according to harmonized guidelines using fertility-controlled culture media that help to standardize the isolate collection process between laboratories belonging to the network [S5]. AST was implemented according to the currently-recommended methods for mycoplasma (7) in the absence of reference method (11) [S6]. Moreover, AST is centralized in one lab, which limits variability in results [S7] but runs counter to the EUCAST recommendations which promote the inclusion of inter-laboratory variability. However, this situation may prove precarious in the longer term [T1], as several methodological choices are done to accommodate time-saving considerations and end up being non-EUCAST compliant (W2), which could introduce AST biases. For example, the choice of one drug per class and only a few concentrations to test could skew interpretation at class scale (32) and thus make it impossible to set ECOFFs. The selection of isolates not initially mixed with other mycoplasma species in culture could also introduce another bias in isolate selection. A transfer of the AST method to voluntary laboratories would allow a multicentre testing approach, although further improvements are still needed to make it routine-compatible [O3]. In contrast, the development of PCR-based clinical diagnosis instead of culture protocols at in-network laboratories could affect AMR surveillance, as isolates would be no longer available in routine [T3].

AST results are reported to stakeholders every year, concomitantly with the Vigimyc clinical surveillance report, which is a recommendations-compliant frequency (6) [S8]. However, for rare or infrequent species, an annual report has only limited interest [W3], so multiannual reporting would be more appropriate. Furthermore, for most species, feedback to veterinarians, which count among the stakeholders, remains limited due to the absence of CBP and thus of clinical interpretation of AST and hence hypotheses about *in vivo* efficacy [W4]. Contribution of Vigimyc AMR surveillance could be considered for ECOFF setting, but remains limited for *M. bovis* as the number of WT isolates was too low for compliance with the EUCAST recommendations [W5]. This difficulty cannot be overcome by increasing the number of tested isolates, as older isolates of the species were already resistant (15, 17). In some cases, such as lincosamides for the *M. mycoides* group, the WT population was barely distinguishable from the non-WT population that was spread across a wide range of MICs. Nonetheless, some of our data allowed to set “national” TECOFFs [O4] that could be used to assess the efficacy of AMR control measures. So far, in the absence of harmonized AST methods, data aggregation with other laboratories, worldwide, remains limited [T2].

## Conclusion

AMR surveillance was successfully integrated into the clinical-dedicated Vigimyc network and enabled us to track resistance trends in different pathogenic mycoplasma species in a way that meets most criteria for surveillance guidelines and that link into clinical evolution. This new AMR surveillance framework further benefited from the multi-species and broad-geographical coverage inherent to Vigimyc. This monitoring system meets the needs for surveillance in a context of concern around AMR where mycoplasmas have so far been neglected despite being significant drivers of antimicrobial use. However, the perspectives for a larger-scale contribution of Vigimyc to surveillance remain limited as of now. Our methodological choices mean that our data was only partially compliant with EUCAST requirements, and aggregation with other datasets for ECOFF setting was limited. The absence of clinical interpretative criteria for veterinary mycoplasmas is a bottleneck for therapy guidance, which is nevertheless the primary objective of AST. Method development and interpretation guidelines should be made a priority for the next series of developments on mycoplasma AMR.

## Data Availability Statement

The reference MIC values and distributions of recent and older populations of *M. mycoides* subsp. *capri, M. capricolum subsp. capricolum* and *M. putrefaciens* are included in the article/Supplementary Material, further inquiries can be directed to the corresponding author/s.

## Author Contributions

MJ and FT designed the study, performed data analysis and wrote the manuscript. FP established *M. mycoides* group reference dataset. AC and AT carried out cultures, strain identification, preservation and AST. All authors read and approved the manuscript content.

## Conflict of Interest

The authors declare that the research was conducted in the absence of any commercial or financial relationships that could be construed as a potential conflict of interest.

## Footnotes

^1^https://agriculture.gouv.fr/plan-ecoantibio-baisse-de-37-de-lexposition-des-animaux-aux-antibiotiques

^2^https://agreste.agriculture.gouv.fr/agreste-saiku/?plugin=true&query=query/open/G_1010#query/open/G_1010

## Abbreviations

AMR: antimicrobial resistance
AST: antimicrobial susceptibility testing
CBP: clinical breakpoint
CLSI: Clinical & Laboratory Standards Institute
ECOFF: epidemiological cut-off value
TECOFF: tentative ECOFF
EUCAST: European Committee on Antimicrobial Susceptibility Testing
MIC: minimum inhibitory concentration
SWOT: Strengths-Weaknesses-Opportunities-Threats
WT: wild-type.
