# Supplementary figures and images for: Addressing the Antimicrobial Resistance of Ruminant Mycoplasmas Using a Clinical Surveillance Network
Source: Front Vet Sci. 2021 Jun 14;8:667175. doi: 10.3389/fvets.2021.667175 (PMC8236625; doi:10.3389/fvets.2021.667175)

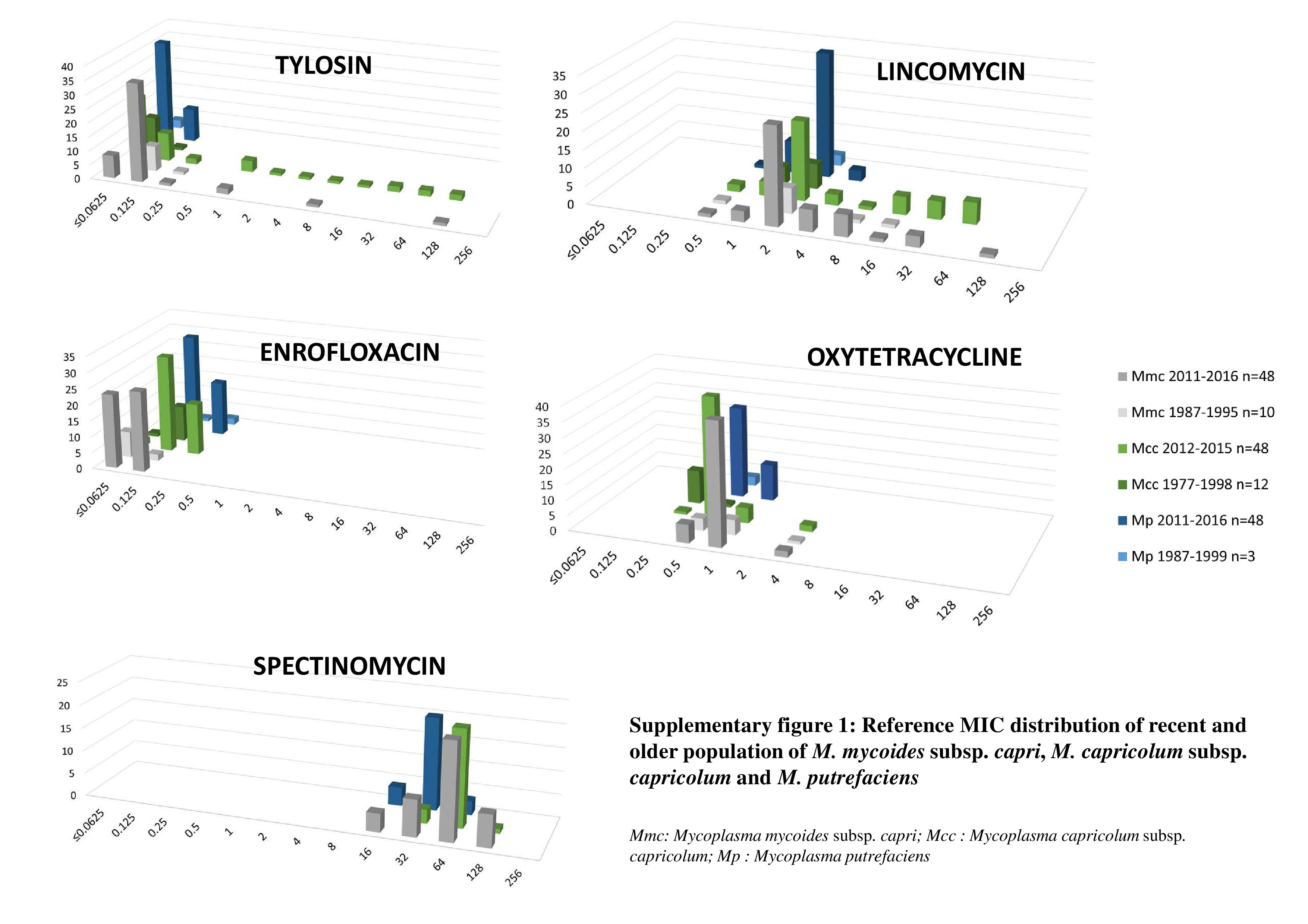

Supplement: Supplementary file 2 [file Image_1.JPEG]
